# Quality of Chronic Obstructive Pulmonary Disease Information on the Chinese Internet: Website Evaluation Study

**DOI:** 10.2196/56594

**Published:** 2024-08-01

**Authors:** Qinqin Wang, Lingjun Liu, Hong Li, Qiao Zhang, Qianli Ma

**Affiliations:** 1 Chronic Respiratory Disease Management and Rehabilitation Center SongShan General Hospital Chongqing China

**Keywords:** chronic obstructive pulmonary disease, internet, information quality, DISCERN, websites, health information, DISCERN instrument, pulmonary disease, chronic pulmonary disease, cross-sectional study, website information, treatment, COPD, China, evaluation, pulmonary, chronic

## Abstract

**Background:**

The development of internet technology has greatly increased the ability of patients with chronic obstructive pulmonary disease (COPD) to obtain health information, giving patients more initiative in the patient-physician decision-making process. However, concerns about the quality of website health information will affect the enthusiasm of patients’ website search behavior. Therefore, it is necessary to evaluate the current situation of Chinese internet information on COPD.

**Objective:**

This study aims to evaluate the quality of COPD treatment information on the Chinese internet.

**Methods:**

Using the standard disease name “慢性阻塞性肺疾病” (“chronic obstructive pulmonary disease” in Chinese) and the commonly used public search terms “慢阻肺” (“COPD”) and “肺气肿” (“emphysema”) combined with the keyword “治疗” (“treatment”), we searched the PC client web page of Baidu, Sogou, and 360 search engines and screened the first 50 links of the website from July to August 2021. The language was restricted to Chinese for all the websites. The DISCERN tool was used to evaluate the websites.

**Results:**

A total of 96 websites were included and analyzed. The mean overall DISCERN score for all websites was 30.4 (SD 10.3; range 17.3-58.7; low quality), no website reached the maximum DISCERN score of 75, and the mean score for each item was 2.0 (SD 0.7; range 1.2-3.9). There were significant differences in mean DISCERN scores between terms, with “chronic obstructive pulmonary disease” having the highest mean score.

**Conclusions:**

The quality of COPD information on the Chinese internet is poor, which is mainly reflected in the low reliability and relevance of COPD treatment information, which can easily lead consumers to make inappropriate treatment choices. The term “chronic obstructive pulmonary disease” has the highest DISCERN score among commonly used disease search terms. It is recommended that consumers use standard disease names when searching for website information, as the information obtained is relatively reliable.

## Introduction

### Background

The internet is an increasingly important source of health information for the public [1,2]. According to data from the China Internet Network Information Center, the number of website medical users in China is nearly 300 million [3]. In the internet era, the public will have an unprecedented ability to obtain health information. This change in the ability to obtain information gives patients more initiative in the process of shared decision-making between doctors and patients. It has become a catalyst for the transformation of the doctor-patient relationship from an autocratic, paternalistic model to a collaborative, shared decision-making model [4]. Ideally, consumers should be able to obtain effective and relevant information about their health status whenever they wish and evaluate all possible actions and their pros and cons according to their values, beliefs, preferences, and personal circumstances. However, the reality is that finding the information you want on the internet is often time-consuming. Consumers are often confused and anxious by the unlimited amount of information available, which is poorly organized and of varying quality and relevance [5].

According to the definition of health information in the eHealth Code of Ethics, health information includes information for staying well, preventing and managing disease, and making other decisions related to health and health care. It includes information for making decisions about health products and health services [6]. Information quality (IQ) refers to the extent to which information is fit for a specific purpose [7]. IQ is multidimensional, with each dimension describing a unique aspect of the information. The common elements of the IQ framework include 7 dimensions: temporal dimension, accessibility or obtainability, objectivity, relevance, accuracy, consistency, and completeness [8].

In order to comprehensively evaluate the various dimensions of IQ, many foreign internet medical IQ evaluation tools have conducted IQ-oriented internet medical evaluation services to select high-quality and valuable medical information for users. There are 3 main mechanisms for evaluating the quality of internet health information: codes of conduct or ethics, third-party certification, and tool-based evaluation [9]. Most of the above internet medical IQ evaluation tools take English information resources as the evaluation object, and because of the high cost of research and development and operation of the evaluation mechanism of code of conduct and third-party certification, as a result, some evaluation tools are limited to domestic network information resources. The level of internet construction in China is still unable to meet the conditions for the use of these 2 types of evaluation tools.

Tool-based evaluation scales have relatively wide applicability because of their ease of use, high consistency of the process used by users, no limitation on the development of internet technology, and no cost of daily maintenance. It is reported that DISCERN is the first scale for users to participate in the evaluation of website medical information resources [10]. Reviewing previous studies, the Chinese website information evaluation mainly focuses on using foreign information evaluation tools to evaluate domestic-specific health websites, and the disease information is limited to lung cancer, cervical cancer, breast cancer, etc [11-13]. The evaluation results of these diseases showed that the quality of health information on Chinese websites was poor.

Chronic obstructive pulmonary disease (COPD) is now one of the top 3 leading causes of death worldwide [14]. In the Chinese general population older than 20 years, the overall incidence of COPD is 8.6%, and the number of patients is approximately 100 million [15]. COPD is characterized by airflow limitation, long disease duration, and persistent dyspnea, which severely affects patients’ daily lives [16]. Although chronic respiratory diseases are not curable, by gaining knowledge and skills about the disease, patients with COPD can actively participate in the self-management of the disease, which can effectively improve their daily activities and quality of life [17,18]. One study found that 41.8% of patients with chronic diseases would use the internet to search for disease-related knowledge [19], but concerns about the quality of website health information often have a negative impact on patients’ search behavior [20,21]. At the same time, limited by the patient’s personal health literacy level, the health information on the internet may be misleading or misunderstood, which may affect health behavior and health outcomes [22,23].

As one of the most common chronic respiratory diseases, the treatment information for COPD on Chinese websites has not received much attention. This study will use DISCERN to evaluate the quality of Chinese COPD treatment information websites and explore factors that may affect the quality of patient access to health information.

### Objective

After an extensive review of the literature on website evaluation, to the best of our knowledge, this is the first study to evaluate the quality of Chinese internet information related to treatment options for COPD.

Given the high prevalence of COPD worldwide, the involvement of patients and caregivers in disease management, and the impact of website information on shared decision-making, we believe that evaluating the quality of websites providing COPD treatment information is the primary objective of this study.

The secondary aim of this study is to explore the factors that may affect the quality of patients’ access to COPD treatment information on Chinese websites and to provide evidence for patients to screen for high-quality COPD treatment information.

## Methods

### Website Selection—Search Strategy and Data Collection

The keywords selected were based on a published study by our team on the public’s search behavior on COPD. In the previous study, our team used the Baidu Index big data analysis tool from January 1, 2011, to December 31, 2018, as the time range, with “慢阻肺” (“COPD” in Chinese), “慢性阻塞性肺疾病” (“chronic obstructive pulmonary disease”), “肺气肿” (“emphysema”), “慢性支气管炎” (“chronic bronchitis”), and “COPD” (“English abbreviation of disease name”) as 5 keywords. We recorded the “search index” and “media index” data on a weekly basis, summarized them quarterly, and generated the data for secondary analysis. The results showed that the search index for the keywords “emphysema” and “COPD” was significantly higher than other keywords (*P*<.001) [24]. The feature of this study is that the observation period is long, and netizens’ search habits are counted directly from the network backend, which avoids sampling errors in qualitative research due to sampling surveys. Therefore, “emphysema,” “COPD,” and the disease standard name “chronic obstructive pulmonary disease” were selected as the 3 core terms for this study. Baidu, Sogou, and 360 search engines were selected because they were among the top 3 search engines in mainland China in terms of brand penetration.

In total, 3 keywords “慢性阻塞性肺疾病” (“chronic obstructive pulmonary disease” in Chinese), “慢阻肺” (“COPD”), and “肺气肿” (“emphysema”) combined with the keyword “治疗” (“treatment”) were searched in 3 search engines (Baidu, Sogou, and 360) from July to August 2021. The incognito window on Microsoft Edge was used to conduct the search, and the browser history, cache, and cookies were cleared before the search to ensure that previous searches would not affect the search results. As most internet users do not search beyond the first 50 sites, we only examined the first 50 sites per search engine [25]. The inclusion and exclusion criteria are presented in Textbox 1.

The 2 authors (QW and LL) independently determined which of the websites met the inclusion criteria. Disagreements were discussed and a third author (HL) was consulted when further discussion was required.

**Inclusion and exclusion criteria.**

**Inclusion criteria**

- Websites (in Chinese language) that provide health information related to chronic obstructive pulmonary disease treatment

**Exclusion criteria**

- Duplicate websites
- Websites that require registration or payment or are inaccessible
- Websites marked as “advertisements”
- Papers or courseware for professional
- Image, audio, and video websites
- Jump link: as most websites contain links to additional resources, we only evaluate the main website, and exclude jump links outside the main website
- Book: only the book introduction website
- website consultation
- Non-Chinese website
- Wikipedia media pages
- News reports
- Blogs or private web

### Procedure

#### Measures of Website Reliability

As we aimed to evaluate sites from the perspective of the patient and the patient’s family, we evaluated all the evaluable websites using the DISCERN instrument. The DISCERN instrument is a validated 16-item questionnaire that emphasizes the reliability of information, focuses on the intrinsic characteristics of health information, and pays more attention to specific aspects. For example, it requires websites to describe the mechanism of action, efficacy, and risks of treatment options, explain other possible treatment options, their impact on quality of life, and what would happen if left untreated [10]. As a specific tool for evaluating disease treatment options, DISCERN has been used in several health website evaluation studies [26,27]. The reliability of the DISCERN has been assessed in previous research, and it has been shown to discriminate between low- and high-quality information [28-31]. The validity and reliability of the translated Chinese version of the DISCERN have been shown to be able to assess the quality of information provided to patients about their choice of treatment [32]. DISCERN consists of 3 main sections focusing on the reliability of the publication (items 1-8), the quality details of information for treatment choices (items 9-15), and the overall quality of the publication (item 16). A series of 15 questions (Table 1) are asked about the content, and each item is rated on a scale of 1 to 5, with 1 indicating that the criterion was not met at all, 2-4 indicating that the criterion was met to some extent, and 5 indicating that the criterion was met completely.

After reviewing and discussing the DISCERN handbook, 2 authors (QW and LL) rated all the included websites. Interrater agreement was calculated.

**Table 1:** Overall mean and standard deviations for the items of the DISCERN quality criteria for the 96 included websitesa.

	DISCERN items (Charnock et al [10])	mean (SD; 95% CI)
1	Are the aims clear?	3.4 (1.2; 3.3-3.5)
2	Does it achieve its aims?	2.7 (1.4; 2.6-2.9)
3	Is it relevant?	2.7 (1.4; 2.6-2.9)
4	Is it clear what sources of information were used to compile the publication?	1.3 (1.0; 1.2-1.5)
5	Is it clear when the information used or reported in the publication was produced?	1.4 (1.0; 1.2-1.5)
6	Is it balanced and unbiased?	2.9 (1.6; 2.7-3.0)
7	Does it provide details of additional sources of support and information?	1.4 (0.9; 1.3-1.5)
8	Does it refer to areas of uncertainty?	1.4 (0.8; 1.3-1.5)
9	Does it describe how each treatment works?	2.2 (1.3; 2.1-2.4)
10	Does it describe the benefits of each treatment?	2.3 (1.2; 2.1-2.4)
11	Does it describe the risks of each treatment?	1.5 (1.0; 1.4-1.6)
12	Does it describe what would happen if no treatment were used?	1.6 (1.1; 1.4-1.7)
13	Does it describe how the treatment choices affect the overall quality of life?	1.7 (0.9; 1.6-1.8)
14	Is it clear that there may be more than one possible treatment choice?	2.5 (1.4; 2.4-2.7)
15	Does it provide support for shared decision-making?	1.5 (0.9; 1.4-1.6)
16	Based on the answers to all of the above questions, rate the overall quality of the publication as a source of information about treatment choices.	2.2 (1.3; 2.0-2.3)

^a^Each DISCERN item is rated on a 5-point scale, anchored at 1=the criterion was not met at all and 5=the criterion was fully met.

#### Analyses

Descriptive analyses provided a mean score for each website and a mean score for each DISCERN item. Pearson correlation was used to test the consistency between 2 raters (QW and LL). The Kruskal-Wallis test was used to detect differences in the mean DISCERN score between the 3 search engines and 3 search items. The significance level was set at *P*<.05. All the statistics were calculated by using SPSS (version 22.0; IBM Corp).

### Ethical Considerations

This study was excluded from institutional ethics board review, as the Chongqing Songshan General Hospital Institutional Review Board does not review studies that do not involve human participants.

## Results

### Search Results

A total of 450 websites were identified using 3 search engines searching for 3 keywords (3 search engines×3 keywords×50 first websites). After applying the exclusion criteria, 96 unique websites were included in the evaluation, 46 websites from Baidu, 28 websites from Sogou, and 22 websites from 360 (Multimedia Appendix 1).

### Website Characteristics

Table 2 presents the selection of websites.

**Table 2:** Selection of websites (frequency distribution).

Initial search for websites		Baidu (n=150), n (%)	Sougou (n=150), n (%)	360 (n=150), n (%)	Total (N=450), n (%)
**Excluded**					
	Advertisement	18 (4)	47 (10.4)	20 (4.4)	85 (18.9)
	website consultation	58 (12.9)	54 (12)	44 (9.8)	156 (34.7)
	Video or audio or image	5 (1.1)	3 (0.7)	13 (2.9)	21 (4.7)
	Jump link	5 (1.1)	4 (0.9)	13 (2.9)	22 (4.9)
	Duplicate	10 (2.2)	10 (2.2)	12 (2.7)	32 (7.1)
	Paid or registered	5 (1.1)	0 (0)	5 (1.1)	10 (2.2)
	Inaccessible	0 (0)	1 (0.2)	2 (0.4)	3 (0.7)
	Books	2 (0.4)	3 (0.7)	1 (0.2)	6 (1.3)
	Professional	1 (0.2)	0 (0)	18 (4)	19 (4.2)
Selected websites		46 (10.2)	28 (6.2)	22 (4.9)	96 (21.3)

### Website Quality

The DISCERN score for each website was independently evaluated. The interrater reliability (Pearson correlation) for the overall median DISCERN score was 0.737 (*P*<.001).

Using the total DISCERN score from the 15 items, the websites were grouped into categories of excellent (63-75), good (51-62), fair (39-50), poor (27-38), and very poor (15-26) in content. Websites included were categorized by DISCERN score as good (6/96, 6.3%), fair (11/96, 11.5%), poor (36/96, 37.5%), and very poor (43/96, 44.8%). None of the websites were rated as “excellent.” The mean overall DISCERN score was 30.4 (SD 10.3; range 17.3-58.7; poor quality).

The mean scores from all 96 websites for each of the 15 DISCERN items are shown in Table 1. The DISCERN scores vary widely for individual items, with scores ranging from 1.2 to 3.9 out of a total score of 5. No item had a mean score of 4 or more. The mean score of item 16, which served as an overall rating of the websites, was 2.2 (SD 1.0), indicating that the overall quality of websites with information about COPD treatment was poor. The highest DISCERN score was achieved for clearly stating the objectives (mean score of 3.4, SD 1.2).

The lowest scores were for sources of information, timing of information generation, available treatment options, uncertainty and risks of treatment, consequences of nontreatment, impact of treatment choices on quality of life, and recommendations to support shared decision-making.

### Website Comparison

#### Comparison of 3 Search Engines

Comparing the DISCERN scores of the websites from the 3 search engines, Baidu (mean 2.2, SD 0.9) had the highest score, Sougou (mean 2.1, SD 0.7) had the second highest score, and 360 (mean 1.7, SD 0.7) had the lowest score. There were significant differences in the mean DISCERN scores between the 3 search engines (*P*<.001).

#### Comparison of 3 Search Terms

Using the standard disease name “chronic obstructive pulmonary disease” (mean 2.5, SD 0.6) as a search term, the website achieved the highest DISCERN score, followed by the abbreviation of the disease name “COPD” (mean 2.3, SD 0.9), and the term “emphysema” (mean 1.6, SD 0.6) achieved the lowest DISCERN score. There were significant differences in the mean DISCERN scores between the 3 search terms (*P*<.001).

## Discussion

### Principal Findings and Comparison With Prior Work

The primary purpose of this study is to evaluate the quality of COPD treatment information on Chinese websites using DISCERN, and the secondary purpose is to explore factors that may affect the quality of COPD information received by patients on Chinese websites.

Currently, there is a lack of behavioral research on Chinese patients with COPD seeking website health information. According to the results of a website health information search on patients with COPD conducted by Stellefson et al [33], most participants reported using their desktops or laptops to access the internet for health information, rather than using mobile phones. At the same time, the study by Hone et al [34] also found that the proportion of people using search engines to find website health information was much higher than the proportion using apps or social networks, which is consistent with the findings of Connelly et al [35] findings. The reason for this result may be that patients with COPD are mainly older people, and unlike young people who are used to watching embedded videos on websites, older people are more used to using the convenience and anonymity of search engines, that is, patients can obtain a large amount of health information without entering personal information [36]. Therefore, this study was based on consumers’ choices and habits and used search engines as a medium to obtain website health information.

Using 3 search engines (Baidu, Sougou, and 360) with the keywords “chronic obstructive pulmonary disease,” “COPD” and “emphysema,” of the 450 websites searched initially, 34.7% (n=156) were website consultations, and 18.9% (n=85) were marked as “advertisements,” accounting for the highest proportion of search results, which posed the first challenge to obtaining objective and valuable information.

The average total DISCERN score was only 30.4, no website achieved the maximum DISCERN score of 75, and the average score for each of the 15 DISCERN items was 2.0. Previous authors have suggested that the scores of the first 15 items should be divided into the following categories: “excellent” (63-75), “good” (51-62), “fair” (39-50), “poor” (2-38), and “very poor” (15-26) [37-40]. Using these criteria, none of the websites were rated as excellent, only 6.3% (n=6) of the sites were rated as good, while 82.3% (n=79) were rated as poor (n=96, 37.5%) or very poor (n=43, 44.8%). Furthermore, when looking at the average of the overall DISCERN rating (item 16), the quality of the website was below average (2.2/5). This suggests that the reliability, detail, and overall quality of COPD website information was poor, which is consistent with previous studies of website quality ratings for other disease-specific information [41-43].

On the DISCERN items for these evaluation websites, only the first item scored above 3 points (3.4/5), with the other items scoring below 3. The areas with the lowest scores (less than 2) relate to providing information or reference sources, describing the risks of each treatment, mentioning areas of uncertainty, encouraging shared decision-making, the consequences of not taking treatment, and how treatment choices affect the overall quality of life, all of which are key information to help patients make treatment decisions. This finding shows that the lack of detailed information about COPD treatment on Chinese websites can directly affect consumers’ judgment of the reliability of the information. It also reminds information providers to pay attention to the unmet health information needs of consumers, ensure the comprehensiveness and reliability of information content, and ensure that the website is regularly updated to include the latest information.

The average DISCERN score of the “chronic obstructive pulmonary disease” was higher than that of “COPD” and “emphysema” among the 3 search terms. The effect of keywords on website quality can also be seen in the results for other specific diseases. Similar to this study, several studies have reported that websites with higher IQs can be obtained by using more precise medical terms [44,45]. Our research findings suggest that health care providers should encourage patients to use standard disease names rather than user-friendly disease search terms when searching for website information, which is beneficial for obtaining high-quality treatment information with patients.

Whether in terms of information reliability (items 1-8), information details (items 9-15), or overall quality (item 16), the DISCERN score of 360 search engine is significantly lower than that of Baidu and Sogou (Multimedia Appendix 2). Previous studies compared the website IQ of Baidu and Google, and the results showed that the IQ rating of Baidu was significantly lower than that of Google [46]. Although there are differences in the retrieval mechanisms used by different network platforms, and there is a lack of unified implementation standards, providing consumers with high-quality information should be the service goal of all network platforms. It is expected that the government departments, together with medical institutions and internet companies, will work together to make suggestions, draw on the diversified and mature evaluation methods of internet health information abroad, develop workable quality standards suitable for China’s network environment, and provide consumers with high-quality health-related information.

### Limitations and Future Outlook

This study has several limitations. The scope of this study was limited to Chinese-language websites. Websites were excluded if they were non-Chinese; therefore, the results may not be applicable to a non–Chinese-speaking patient population. Another limitation may be related to the selection of search terms. Although the keywords were chosen to represent the most common searches, it is possible that information seekers may use other terms. The use of other keywords could result in the identification of other websites that may have different quality outcomes to those of the websites included in this study.

DISCERN, as a standard for the evaluation of health information, mainly assesses the relevance and reliability of the information on the website, but this is only one aspect of IQ and cannot represent the overall quality of the information, as DISCERN does not consider whether the information provided by the website is scientific and evidence-based. For the team’s future research plan, it is very valuable to investigate specific Chinese websites on COPD in terms of the criteria of understandability and actionability, website credibility, website design, usability, and readability, which is conducive to the comprehensive evaluation of website IQ.

In addition, as the resources available on the internet are constantly growing and changing, search results retrieved at different times may differ. These results can be used as a benchmark for future reassessment.

### Conclusions

This was the first study to evaluate the quality of Chinese-language internet information on COPD treatment. The quality of COPD information on the Chinese internet is poor. Most of the websites reviewed lack a detailed description of the content of health information, including the description of the relevance of the target population at the beginning of the content, as well as the source of support for the information, the reporting time of the information, the support for shared decision-making, the risks and benefits of treatment choices and their impact on quality of life, and the consequences of nontreatment. It is difficult for consumers to obtain detailed health information from the internet, resulting in inappropriate treatment choices. This study also warns that the impact of website IQ on patients’ treatment decisions should attract the attention of network platform regulators, medical service providers, and internet companies. They should develop quality evaluation standards for information production and dissemination to help patients avoid incomplete or misleading websites. Meanwhile, consumers should use standard disease names, which helps to obtain high-quality health information.

## Appendix

Multimedia Appendix 1 Flow diagram of the search results.

Multimedia Appendix 2 DISCERN score of websites obtained by 3 search engines (mean, SD).

## Abbreviations

COPD: chronic obstructive pulmonary disease
IQ: information quality

## References

[1] Eysenbach G (2000). Consumer health informatics. BMJ.

[2] Dumitru RC, Bürkle T, Potapov S, Lausen B, Wiese B, Prokosch H (2007). Use and perception of internet for health related purposes in Germany: results of a national survey. Int J Public Health.

[3] (2022). The 49th statistical report on internet development in China.

[4] Gerber BS, Eiser AR (2001). The patient physician relationship in the internet age: future prospects and the research agenda. J Med Internet Res.

[5] Eysenbach G, Jadad AR (2001). Evidence-based patient choice and consumer health informatics in the internet age. J Med Internet Res.

[6] e-Health Ethics Initiative (2000). e-Health code of ethics (May 24). J Med Internet Res.

[7] Gerkes M (1997). Information quality paradox of the web.

[8] Fadahunsi KP, O'Connor S, Akinlua JT, Wark PA, Gallagher J, Carroll C, Car J, Majeed A, O'Donoghue J (2021). Information quality frameworks for digital health technologies: systematic review. J Med Internet Res.

[9] Risk A, Dzenowagis J (2001). Review of internet health information quality initiatives. J Med Internet Res.

[10] Charnock D, Shepperd S, Needham G, Gann R (1999). DISCERN: an instrument for judging the quality of written consumer health information on treatment choices. J Epidemiol Community Health.

[11] Chao W, Hao W, Bi JQ, Sun YC (2018). Quality evaluation on health information of lung cancer in Chinese websites. China Cancer.

[12] Zhen HM, Miao Z, Lian X, Hu K, Shen J, Ma JB, Guan H, Zhang FQ (2019). Information needs and quality among cervical cancer patients. Zhongguo Yi Xue Ke Xue Yuan Xue Bao.

[13] Li Y, Zhou X, Zhou Y, Mao F, Shen S, Lin Y, Zhang X, Chang T, Sun Q (2021). Evaluation of the quality and readability of online information about breast cancer in China. Patient Educ Couns.

[14] Halpin DMG, Celli BR, Criner GJ, Frith P, Varela MVL, Salvi S, Vogelmeier CF, Chen R, Mortimer K, Montes de Oca M, Aisanov Z, Obaseki D, Decker R, Agusti A (2019). The GOLD Summit on chronic obstructive pulmonary disease in low- and middle-income countries. Int J Tuberc Lung Dis.

[15] Wang C, Xu J, Yang L, Xu Y, Zhang X, Bai C, Kang J, Ran P, Shen H, Wen F, Huang K, Yao W, Sun T, Shan G, Yang T, Lin Y, Wu S, Zhu J, Wang R, Shi Z, Zhao J, Ye X, Song Y, Wang Q, Zhou Y, Ding L, Yang T, Chen Y, Guo Y, Xiao F, Lu Y, Peng X, Zhang B, Xiao D, Chen C, Wang Z, Zhang H, Bu X, Zhang X, An L, Zhang S, Cao Z, Zhan Q, Yang Y, Cao B, Dai H, Liang L, He J (2018). Prevalence and risk factors of chronic obstructive pulmonary disease in China (the China Pulmonary Health [CPH] study): a national cross-sectional study. Lancet.

[16] Global strategy for the diagnosis, management and prevention of chronic obstructive lung disease (2021 report). Global Initiative for Chronic Obstructive Lung Disease (GOLD).

[17] GBD Chronic Respiratory Disease Collaborators (2020). Prevalence and attributable health burden of chronic respiratory diseases, 1990-2017: a systematic analysis for the Global Burden of Disease Study 2017. Lancet Respir Med.

[18] Sadeghi S, Brooks D, Goldstein RS (2013). Patients' and providers' perceptions of the impact of health literacy on communication in pulmonary rehabilitation. Chron Respir Dis.

[19] Juan L (2022). A study on the current situation of electronic health literacy and network health information search behavior of patients with chronic diseases.

[20] Fergus TA, Dolan SL (2014). Problematic internet use and internet searches for medical information: the role of health anxiety. Cyberpsychol Behav Soc Netw.

[21] Moult A, Burroughs H, Kingstone T, Chew-Graham CA (2018). How older adults self-manage distress—does the internet have a role? A qualitative study. BMC Fam Pract.

[22] McClung HJ, Murray RD, Heitlinger LA (1998). The internet as a source for current patient information. Pediatrics.

[23] Kiley R (2002). Does the internet harm health? Some evidence exists that the internet does harm health. BMJ.

[24] Wan M, Li Q, Zhang Q, Ma Q (2020). Public awareness for chronic obstructive pulmonary disease based on baidu index. Chin J Lung Dis (electronic edition).

[25] Eysenbach G, Köhler Christian (2002). How do consumers search for and appraise health information on the world wide web? Qualitative study using focus groups, usability tests, and in-depth interviews. BMJ.

[26] Felipe L, Beukes EW, Fox BA, Manchaiah V (2020). Quality and readability of English-language internet information for vestibular disorders. J Vestib Res.

[27] Da Silva T, Lokhandwala A, Al Kaabi N, Semenchuk J, Goobie GC, Camacho E, Reid WD, Fisher JH, Ryerson CJ, Rozenberg D (2023). Characterization and reliability of internet resources on pulmonary rehabilitation for individuals with chronic lung disease. Chron Respir Dis.

[28] Rees CE, Ford JE, Sheard CE (2002). Evaluating the reliability of DISCERN: a tool for assessing the quality of written patient information on treatment choices. Patient Educ Couns.

[29] Charnock D, Shepperd S (2004). Learning to DISCERN online: applying an appraisal tool to health websites in a workshop setting. Health Educ Res.

[30] Hargrave DR, Hargrave UA, Bouffet E (2006). Quality of health information on the internet in pediatric neuro-oncology. Neuro Oncol.

[31] Pryor TAM, Reynolds KA, Kirby PL, Bernstein MT (2022). Quality of late-life depression information on the internet: website evaluation study. JMIR Form Res.

[32] Shan Y, Xing Z, Dong Z, Ji M, Wang D, Cao X (2023). Translating and adapting the DISCERN instrument into a simplified Chinese version and validating its reliability: development and usability study. J Med Internet Res.

[33] Stellefson ML, Shuster JJ, Chaney BH, Paige SR, Alber JM, Chaney JD, Sriram PS (2018). Web-based health information seeking and eHealth literacy among patients living with chronic obstructive pulmonary disease (COPD). Health Commun.

[34] Hone T, Palladino R, Filippidis FT (2016). Association of searching for health-related information online with self-rated health in the European Union. Eur J Public Health.

[35] Connelly TM, Khan MS, Victory L, Mehmood A, Cooke F (2018). An assessment of the quality and content of information on diverticulitis on the internet. Surgeon.

[36] Hämeen-Anttila K, Pietilä K, Pylkkänen L, Pohjanoksa-Mäntylä M (2018). Internet as a source of medicines information (MI) among frequent internet users. Res Social Adm Pharm.

[37] Salman MY, Bayar G (2021). Evaluation of quality and reliability of YouTube videos on female urinary incontinence. J Gynecol Obstet Hum Reprod.

[38] Ved R, Cobbold N, Igbagiri K, Willis M, Leach P, Zaben M (2017). Online patient information on vagus nerve stimulation: how reliable is it for facilitating shared decision making?. Seizure.

[39] Som R, Gunawardana NP (2012). Internet chemotherapy information is of good quality: assessment with the DISCERN tool. Br J Cancer.

[40] Weil AG, Bojanowski MW, Jamart J, Gustin T, Lévêque M (2014). Evaluation of the quality of information on the Internet available to patients undergoing cervical spine surgery. World Neurosurg.

[41] Aydin MF, Aydin MA (2020). Quality and reliability of information available on YouTube and Google pertaining gastroesophageal reflux disease. Int J Med Inform.

[42] Szmuda T, Özdemir C, Fedorow K, Ali S, Słoniewski P (2021). YouTube as a source of information for narcolepsy: a content-quality and optimization analysis. J Sleep Res.

[43] Doubleday AR, Novin S, Long KL, Schneider DF, Sippel RS, Pitt SC (2021). Online information for treatment for low-risk thyroid cancer: assessment of timeliness, content, quality, and readability. J Cancer Educ.

[44] Kadam-Halani PK, Lee DD, Sammel MD, Arya LA, Andy UU (2019). The quality of health information available on the internet for patients with fecal incontinence. Female Pelvic Med Reconstr Surg.

[45] Solomon ER, Janssen K, Krajewski CM, Barber MD (2015). The quality of health information available on the internet for patients with pelvic organ prolapse. Female Pelvic Med Reconstr Surg.

[46] Chen T, Gentry S, Qiu D, Deng Y, Notley C, Cheng G, Song F (2020). Online information on electronic cigarettes: comparative study of relevant websites from Baidu and Google search engines. J Med Internet Res.

