# Supplementary material for: Quality of Chronic Obstructive Pulmonary Disease Information on the Chinese Internet: Website Evaluation Study
Source: JMIR Form Res. 2024 Aug 1;8:e56594. doi: 10.2196/56594 (PMC11327628; doi:10.2196/56594)
Supplement: Multimedia Appendix 1 [file formative_v8i1e56594_app1.docx]

Included websites (n=96)

*Baidu* :46

*Sogou* :28

*360* :22

Excluded (n=322)

Advertisements: 85

Website Consultation: 156

Video/Audio/Image: 21

Jump link: 22

Paid/Registered: 10

Professional: 19

Cannot access: 3

Book: 6

Unique websites (n=418)

Duplicated (n=32)

Search terms (in Chinese):

Chronic Obstructive Pulmonary disease,

COPD,

Emphysema,

combine with keyword: treatment

Initial search (n=450)

*Baidu* :150

*Sogou* :150

*360* :150
