# Supplementary material for: Quality of Chronic Obstructive Pulmonary Disease Information on the Chinese Internet: Website Evaluation Study
Source: JMIR Form Res. 2024 Aug 1;8:e56594. doi: 10.2196/56594 (PMC11327628; doi:10.2196/56594)
Supplement: Multimedia Appendix 2 [file formative_v8i1e56594_app2.docx]

| Search Engine | DISCERN | DISCERN^a^ | DISCERN^b^ | DISCERN^c^ |
| --- | --- | --- | --- | --- |
| *Baidu* (n=46) | 2.2, 0.9 | 2.3, 1.0 | 2.0, 0.9 | 2.4, 1.3 |
| *Sogou* (n=28) | 2.1, 0.7 | 2.2, 0.7 | 1.9, 0.7 | 2.3, 1.2 |
| *360* (n=22) | 1.7, 0.7 | 1.7, 0.6 | 1.6, 0.8 | 1.7, 1.2 |
| *P* value^a^ | ＜.001 | ＜.001 | ＜.001 | ＜.001 |

^a^Kruskal‒Wallis test

DISCERN: item1-16; DISCERN^a^: item1-8; DISCERN^b^: item 9-15; DISCERN^c^: item 16
